# A novel *COL4A1* gene mutation results in autosomal dominant non-syndromic congenital cataract in a Chinese family

**DOI:** 10.1186/s12881-014-0097-2

**Published:** 2014-08-15

**Authors:** Xin-Yi Xia, Na Li, Xiang Cao, Qiu-Yue Wu, Tian-Fu Li, Cui Zhang, Wei-Wei Li, Ying-Xia Cui, Xiao-Jun Li, Chun-Yan Xue

**Affiliations:** 1 Institute of Laboratory Medicine, Jinling Hospital, Nanjing University School of Medicine, 305 East Zhongshan Road, Nanjing 210002, People’s Republic of China; 2 Department of Neurology, Jinling Hospital, Nanjing University School of Medicine, 305 East Zhongshan Road, Nanjing 210002, People’s Republic of China; 3 Department of Ophthalmology, Jinling Hospital, Nanjing University School of Medicine, 305 East Zhongshan Road, Nanjing 210002, People’s Republic of China

**Keywords:** Type IV collagen, COL4A1, Non-syndromic congenital cataract

## Abstract

**Background:**

Almost one-third of congenital cataracts are primarily autosomal dominant disorders, which are also called autosomal dominant congenital cataract, resulting in blindness and clouding of the lens. The purpose of this study was to identify the disease-causing mutation in a Chinese family affected by bilateral, autosomal dominant congenital cataract.

**Methods:**

The detection of candidate gene mutation and the linkage analysis of microsatellite markers were performed for the known candidate genes. Molecular mapping and cloning of candidate genes were used in all affected family members to screen for potential genetic mutations and the mutation was confirmed by single enzyme digestion.

**Results:**

The proband was diagnosed with isolated, congenital cataract without the typical clinical manifestations of cataract, which include diabetes, porencephaly, sporadic intracerebral hemorrhage, and glomerulopathy. A novel mutation, c.2345 G > C (Gly782Ala), in exon 31 of the *collagen type IV αlpha1* (*COL4A1*) gene, which encodes the collagen alpha-1(IV) chain, was found to be associated with autosomal dominant congenital cataract in a Chinese family. This mutation was not found in unaffected family members or in 200 unrelated controls. Sequence analysis confirmed that the Gly782 amino acid residue is highly conserved.

**Conclusions:**

The novel mutation (c.2345 G > C) of the *COL4A1* gene is the first report of a non-syndromic, autosomal dominant congenital cataract, thereby highlighting the important role of type IV collagen in the physiological and optical properties of the lens.

## Background

Almost one-third of congenital cataracts, also referred to as autosomal dominant congenital cataract (ADCC), are primarily autosomal dominant disorders that result in blindness and clouding of the lens. Such disorders account for 10% of all childhood blindness worldwide. Additionally, there are a few reports of such disorders being inherited in an autosomal recessive or X-linked manner [1]. ADCC has highly variable morphologies (including total, polar, zonular, and capsular) within families and can include multisystem disorders, such as Wolf-Hirschhorn syndrome, SHORT syndrome, Abruzzo-Erickson syndrome [2], and HANAC syndrome [3]. The clinical manifestation of congenital cataract is multi-organs, including myopathy, cerebral hemorrhages, porencephaly, nephropathy, diabetes, etc. In general, non-syndromic, congenital cataracts that are not related to other disorders are rare, having an estimated frequency of 1–6 per 10,000 live births in industrialized countries, with one-third of cases having a positive family history [3],[4] and 5–15 per 10,000 live births in the poorest areas of the world [3],[5]. To date, a series of congenital cataract-associated chromosomal locations have been mapped and over 30 genes have been identified by linkage analysis. Most of these genes include crystalline genes (*CRYAA*, *CRYAB, CRYBB1*, *CRYBB2*, *CRYBB3*, *CYRBA1*, *CRYBA3*, *CRYBA4*, *CRYGA*, *CRYGB*, *CRYGC*, *CRYGD*, and *CRYGS*) [6], membrane transport genes (*MIP*) [7], and gap junction proteins (*GJA3* and *GJA8*) [8]. The remaining known mutations are found in genes encoding growth and transcription factors, such as *HSF4*, *MAF*, *PITX3*, and *PAX6*[9]. However, it was discovered that *COL4A1* gene mutations were associated with ADCC in French families [10],[11], and there were rare reports that the *type IV collagen*, *αlpha1* (*COL4A1*) gene was associated with non-syndromic, autosomal dominant congenital cataract.

*COL4A1* (NM_001845) and *COL4A2* (NM_001846) encode type IV collagen, which is present in almost all basement membranes and is highly conserved across species, and comprise 52 and 48 exons, respectively. They are arranged head-to-head on opposite strands of human chromosome 13. They are separated by 127 nucleotides containing a shared, bi-directional promoter that requires additional elements to control the tissue specificity and the level of protein expression [12]. Type IV collagen contains three major domains: an amino-terminal 7S domain, which participates in inter-molecular cross-linking and macromolecular organization, and a highly conserved, central triple-helix forming domain and a carboxyl-terminal, non-collagenous domain, which are globular domains responsible for the initiation of heterotrimer assembly [13].

It is known that humans carrying mutations in the *COL4A1* locus often exhibit lens abnormalities and cataracts, along with porencephaly, diabetes, sporadic intracerebral hemorrhage and glomerulopathy [6]. However, a mutation of *COL4A1* gene resulting in isolated congenital cataract has never been reported previously.

## Methods

### Ethics statement

The Ethics Committee of Jinling Hospital approved the protocols used in this study. The research adhered to the tenets of the Declaration of Helsinki. All participants gave written consent to participate in the study, including consent to publish any accompanying images. Parental consent was obtained for children under the age of 16 years old.

### Participant and clinical data

**Figure 1:**
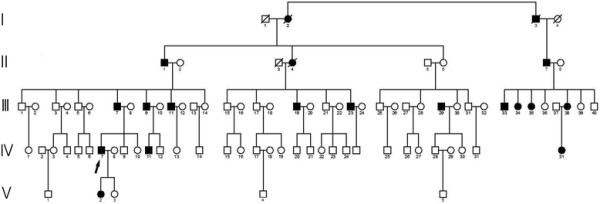
**The pedigree of a five-generation Chinese family with autosomal dominant congenital cataract is shown.** The proband (IV-7) is indicated by a black arrow, and filled symbols indicate individuals with clinical manifestations of congenital cataract. All of the affected individuals carry the same *COL4A1* mutation.

The large pedigree (Figure 1) of a five-generation Han family from a rural area in Jiangsu Province in China includes 15 affected and 64 unaffected individuals with typical features of congenital cataract. The proband (IV-7) came to our hospital for genetic counseling regarding cataract. All living members of this family underwent a systematic physical inspection and an examination that included slit-lamp microscopy of the lens and brain magnetic resonance imaging (MRI).

### Detection of mutation of candidate genes and linkage analysis of microsatellite markers

Seventy-nine cases of the families were studied for linkage of the reported 17 candidate genes and 12 regions of chromosome with haplotypes associated with congenital cataracts using 27 microsatellite markers. However, all selected microsatellite markers were not linked with the reported disease genes and the LOD scores were not meaningful. This suggests that a novel gene mutation may result in congenital cataract.

### Genome-wide genotyping for linkage analysis

A genome-wide linkage scan of the family was conducted to determine the chromosomal regions linked to congenital cataract. Seventy-nine family members (15 affected) participated in this study. DNA samples were genotyped using the Affymetrix GeneChip Human Mapping 500 K Array(Affymetrix, Santa Clara, CA, USA), which contains more than 500,000 SNPs. Array experiments were conducted according to the manufacturer’s protocol. The Affymetrix GeneChip Operating Software (GCOS) was used for image processing. Genotypes were categorized with the Affymetrix Genotyping Console Software (GTC 4.0). Parametric, multipoint linkage analysis was performed using Merlin software under the assumption of autosomal-dominant inheritance with 99% penetrance, a disease allele frequency of 0.1%, and an equal SNP allele frequency (50%).

### DNA sequencing analysis of the *COL4A1* and *COL4A2* genes and enzyme digestion detections

It was shown that six candidate genes, *LIG4*, *MYR8*, *ISR2*, *ING*, *COL4A1*, and *COL4A2*, might be associated with congenital cataracts. According to the instructions, all samples were stored at −20°C. The primers were designed using Primer 5 software based on the sequences of the 53 exons and 48 exons of the *COL4A1* and *COL4A2* genes, respectively, as well as their exon-intron boundaries. Polymerase chain reactions (PCRs) were performed were performed under the following conditions: 95°C for 5 min followed by 35 cycles of 94°C for 30 s, 56°C-60°C for 30 s, and 72°C for 60s, and the products were then sequenced. The sequencing results were compared to those in the NCBI Reference Sequence database. The PCR products from the *COL4A1* gene were detected by enzyme digestion with the endonuclease *Pvu*II.

## Results

### Clinical findings

**Figure 2:**
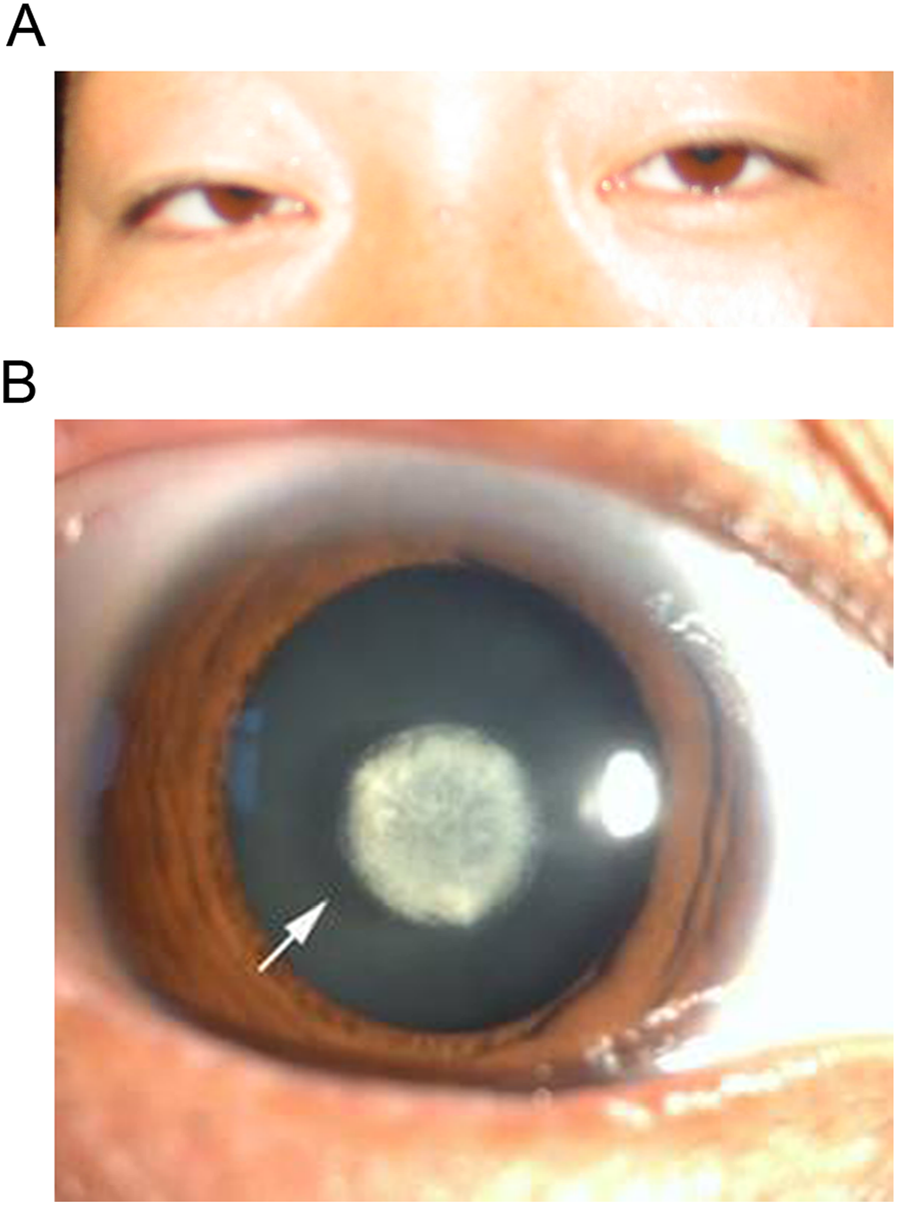
**The slit-lamp photograph of the dilated eye of the proband. (A)** Slit-lamp photograph of the lens of the proband showing the nuclear cataract. All the affected individuals in the family have the same manifestations of nuclear cataract. **(B)** Photograph taken of the proband indicating a normal cornea and iris: a nuclear cataract was present.

The proband (IV-7) came to our hospital for genetic counseling for congenital nuclear cataract, which resulted in blurred vision. The disc-shaped turbidity of the lens was located in the pupil area, as assessed using a slit-lamp (Figure 2A). Brain MRIs and renal function of the affected members were normal. A slit-lamp photograph taken of the proband indicated a normal cornea and iris, as well as the presence of a nuclear cataract (Figure 2B). There were no typical clinical manifestations of cataracts, which include diabetes, porencephaly, sporadic intracerebral hemorrhage, and glomerulopathy. Upon examination, the proband had a normal head posture, with a symmetrical facial appearance and normal dentition. It was further confirmed that the congenital cataract of the family is an autosomal dominant disorder.

### Linkage analysis

Twenty-seven microsatellite markers, according to genes that were previously shown to be associated with congenital cataracts, e.g. *CRYAA*, *CRYAB*, etc., were collected to conduct a linkage analysis of the susceptibility gene of the family. It was revealed that two-point LOD scores were less than minus 2. In other words, the susceptibility of the pedigree was not linked with the previously reported 19 candidate genes and 12 chromosome regions associated with ADCC. Thus, mutation of a novel gene resulted in the congenital cataract of this family.

### Genome-wide genotyping for linkage analysis

**Figure 3:**
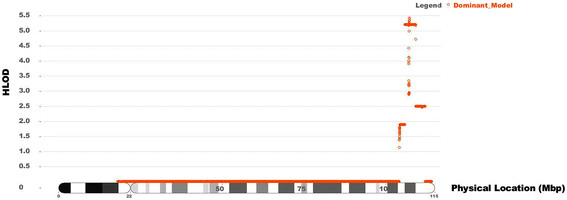
**Parametric linkage analysis results for chromosome 13.** The genetic linkage region spanned approximately 3.3 Mb with a HLOD score of 5.413 on chromosome 13q33.3-q33.4. No significant linkage with markers on other chromosomal regions was identified in the ADCC family.

Parametric, multipoint linkage analysis of the family revealed a genetic linkage region on chromosome 13q33.3-q33.4 (Figure 3). The genetic linkage region spanned approximately 3.3 Mb with a HLOD score of 5.413, and no significant linkage with markers on other chromosomal regions was identified in the ADCC family. Six candidate genes, *LIG6*, *MYR8*, *IRS2, COL4A1*, *COL4A2*, and *ING*, were located within the linkage region.

### Mutational analysis of the *COL4A1* gene and enzyme digestion detections

**Figure 4:**
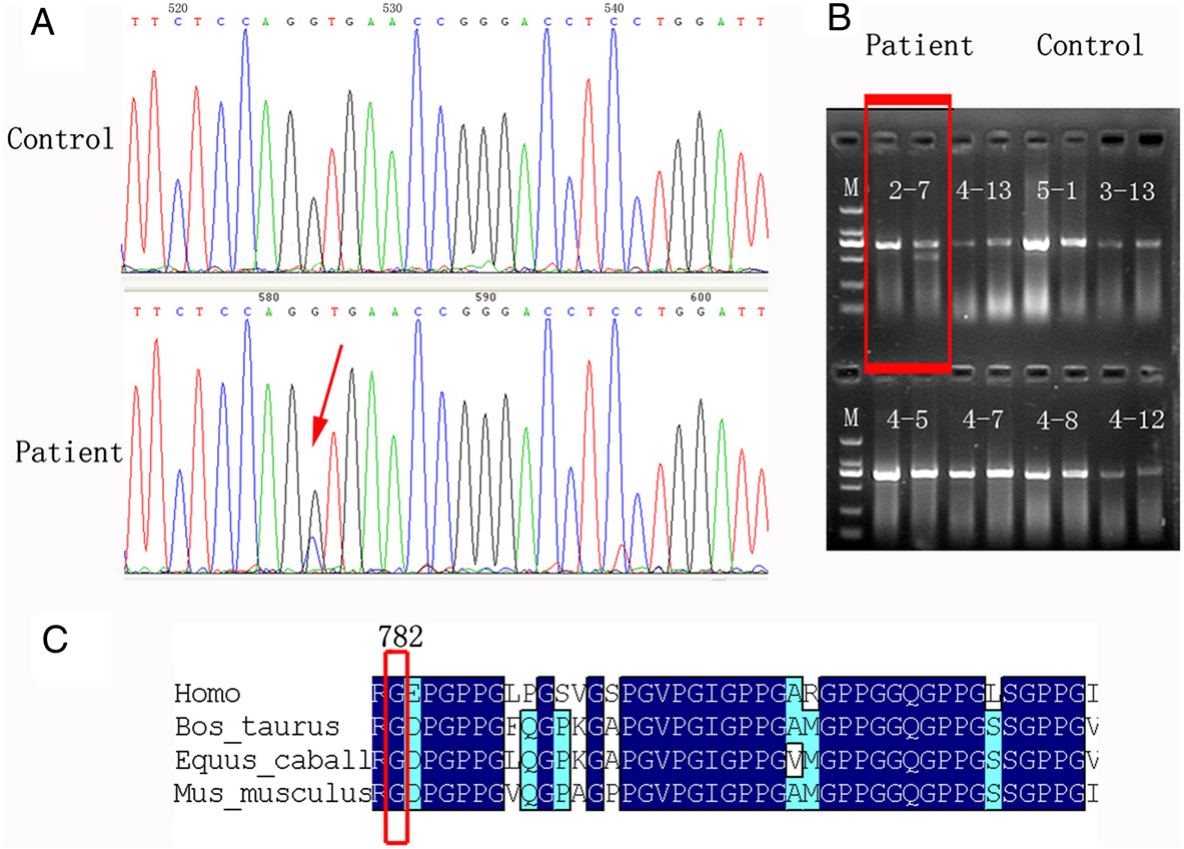
**Sequence analysis of exon 31 of the*COL4A1*gene and protein sequence alignments. (A)** Identification of a *COL4A1* substitution mutation in a Chinese family. Directed sequence analysis of affected individuals revealed a substitution mutation (c.2345 G > C) in exon 31 of the *COL4A1* gene that was not found in 200 control patients. **(B)** Identification of the mutation (c.2345 G > C) carrier by single enzyme digestion with the restriction endonuclease *Pvu*II*.* PCR product amplified from the uncleaved fragment is shown in the first lane and the second lane. **(C)** Protein sequence alignments of multiple species, *Homo sapiens*, *Bos taurus*, *Equus caballus* and *Mus musculus*, indicate a very strong degree of conservation of the amino acid altered by the mutation.

It was reported that *COL4A1* or *COL4A2* mutations can cause ocular, cerebral, renal, and muscular defects [13]. Primers were designed to amplify the exons of *COL4A1* and *COL4A2* and PCRs were performed. The entire *COL4A1* gene was sequenced and a heterozygous G-to-C transition (c.2345 G > C) was identified in exon 31, leading to the replacement of a highly conserved glycine residue by alanine at position 782 (Gly782Ala) within the triple-helix domain (Figure 4C). This mutation, which was not previously described, was not found in a panel of 200 control chromosomes of ethnically matched controls. The mutation was further confirmed by single enzyme digestion with the restriction endonuclease *Pvu*II (Figure 4B).

## Discussion

*COL4* contain six known genes (α1-α6). It was discovered that overexpression of *COL4A3* or *COL4A4* in the embryonic lens results in microphthalmia and cataract [14], and mutation in *COL4A3*, *COL4A3*, and *COL4A5* cause Alport syndrome [15]. The protein products of *COL4A1* and *COL4A2* are present in almoxst all basements membranes of the extracellular matrix (ECM), whereas those of *COL4A3* through *COL4A6* are more spatially and temporally restricted. Congenital or early onset of cataract has previously been observed in patients with mutations in *COL4A1*[16],[17], whereas congenital cataracts were always associated with multi-system disorders, along with cerebrovascular disease (brain small vessel disease and intracerebral hemorrhage), nephropathy, muscle cramps, and ocular anomalies. However, non-syndromic congenital cataracts, namely those not associated with other disorders are rare. Here, we discovered a Gly substitute mutation (c.2345 G > C) in exon 31 of the *COL4A1* gene that results in non-syndromic, autosomal dominant, congenital cataract. This mutation was predicted to have a deleterious effect on protein function by SIFT and Polyphen software(in Additional file 1).

The type IV collagens encoded by the paralogous genes *COL4A1* and *COL4A2* form heterotrimers in vivo: α1α2α3 consists of long stretches of (Gly-X-Y)_n_ repeats, where X and Y are variable amino acids, with proline often occupying the Y position. There also exists an amino-terminal, 7S domain and a carboxyl-terminal, non-collagenous (NC1) domain. Gly in the triple helix domain is highly conserved across species, and mutation of this site may result in the destruction of the triple helix domain of the type IV collagens. Misfolded collagen proteins may, thus, disrupt the integrity of basement membranes in most parts of the eyes. In addition, they may also contribute to cataract development.

*COL4A1* and *COL4A2* are translated at the rough endoplasmic reticulum (ER), where nascent peptides interact with ER resident proteins to ensure proper folding, post-translational modification, and heterotrimer assembly. The Gly substitution mutation of the *COL4A1* gene may result in the accumulation of unfolded, collagenous protein in the ER, and there were reports that this accumulation has been found to cause ER stress in some tissues, resulting in the subsequent activation of the unfolded protein response (UPR). The ER attempts to relieve stress in three ways. The first is to reduce the synthesis of related proteins, the second is to up-regulate the folding capacity of the ER, the last one is to increase the clearance of unfolded proteins [6]. If these mechanisms cannot alleviate the stress, the UPR pathway activates apoptosis. In conclusion, UPR activation in the lens secretary pathway might disrupt lens differentiation and cell survival, resulting in pathologies that lead to cataract formation. Until now, the precise pathologic mechanism resulting from *COL4A1* mutations in patients was poorly characterized and it was only presumed to impair protein secretion, thereby resulting in the intracellular accumulation of misfolded protein in the ER and the subsequent induction of the UPR pathway.

The tissue distribution and pathology severity depend on genetic and environmental factors, which commonly include cerebrovascular diseases, and ocular and renal defects. The *COL4A1* gene is the major element of basement membrane in the ECM and is distributed in all tissues. Mutations in *COL4A1* were first associated with cerebral microangiopathy and familial porencephaly [18]. Several authors have reported that mutations in *COL4A1* may be the Mendelian cause of prenatal onset intracranial hemorrhage [19]. The observed phenotypes are associated with generalized basement membrane (BM) defects, but show a high degree of tissue-specific variability. The non-syndromic, congenital cataract in our report is rare, and the pathological mechanism needs further intensive studies. In summary, our study reports, for the first time, that a *COL4A1* mutation is associated with autosomal dominant, congenital cataract in humans.

## Conclusions

In this study, a novel mutation (c.2345 G > C) of *COL4A1* was detected in a Chinese family, and this mutation extends the mutational spectrum of ADCC. The molecular findings of non-syndromic ADCC resulting from the *COL4A1* mutation highlight the importance of analyzing type IV collagen genes (*COL4A1* and *COL4A2*) in congenital cataract patients.

## Abbreviations

ADCC: Autosomal dominant congenital cataract

COL4A1: Collagen, type IV, αlpha1

CRYAA: Crystallin, alpha A

CRYAB: Crystallin, alpha B

CRYBB1: Crystallin, beta B1

CRYBB2: Crystallin, beta B2

CRYBB3: Crystallin, beta B3

CRYBA1: Crystallin, beta A1

CRYBA2: Crystallin, beta A2

CRYBA3: Crystallin, betaA3

CRYBA4: Crystallin, beta A4

CRYGA: Crystallin, gamma A

CRYGB: Crystallin, gamma B

CRYGC: Crystallin, gamma C

CRYGD: Crystallin, gamma D

CRYGS: Crystallin, gamma S

GJA3: Gap junction protein, alpha 3

GJA8: Gap junction protein, alpha 8

MIP: Major intrinsic protein of lens fiber

MAF: v-maf avian musculoaponeurotic fibrosarcoma oncogene homolog

PITX3: Paired-like homeodomain 3

HSF4: Heat shock transcription factor 4

PAX6: Paired box 6

PCR: Polymerase chain reaction

COL4A1: Type IV collagen, αlpha1

COL4A2: Type IV collagen, αlpha2

COL4A3: Type IV collagen, αlpha3

COL4A4: Type IV collagen, αlpha4

COL4A5: Type IV collagen, αlpha5

MRI: Magnetic resonance imaging

LIG4: Ligase IV

ECM: Extracellular matrixc

NG1: Non-collagenous

ER: Endoplasmic reticulum

UPR: Unfolded protein response

BM: Basement membrane

## Competing interest

The authors declare that they have no competing interests.

## Authors’ contributions

CYX, XYX, NL conducted the experimental work. XYX, NL, XC, QYW, TFL, CZ and WWL analyzed the data. XYX, NL wrote the paper. XJL and YXC provided input for the paper. All authors read and approved the final manuscript.

## Additional file

## Supplementary Material

Additional file 1: Figure S1. The analysis result of SIFT Software. **Figure S2.** The analysis result of Polyphen Software. Click here for file
