# Supplementary material for: A novel COL4A1 gene mutation results in autosomal dominant non-syndromic congenital cataract in a Chinese family
Source: BMC Med Genet. 2014 Aug 15;15:97. doi: 10.1186/s12881-014-0097-2 (PMC4236509; doi:10.1186/s12881-014-0097-2)
Supplement: Additional file 1: Figure S1. — The analysis result of SIFT Software. Figure S2. The analysis result of Polyphen Software. [file s12881-014-0097-2-S1.doc]

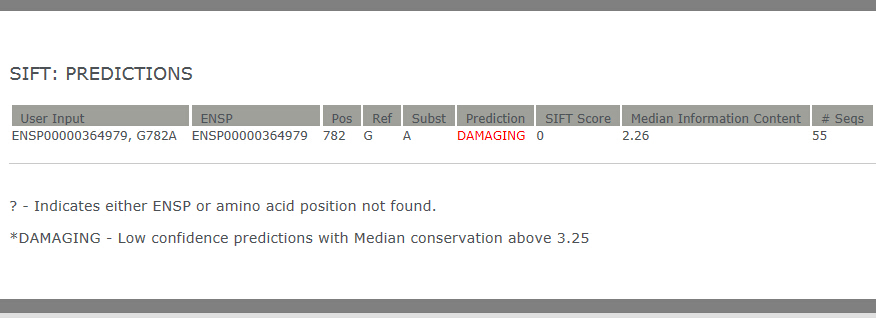
The mutation c.2345 G>C (Gly782Ala) was predicted to have a deleterious effect on protein function by SIFT, Polyphen Software shown in Figure.1 and Figure. 2 below.


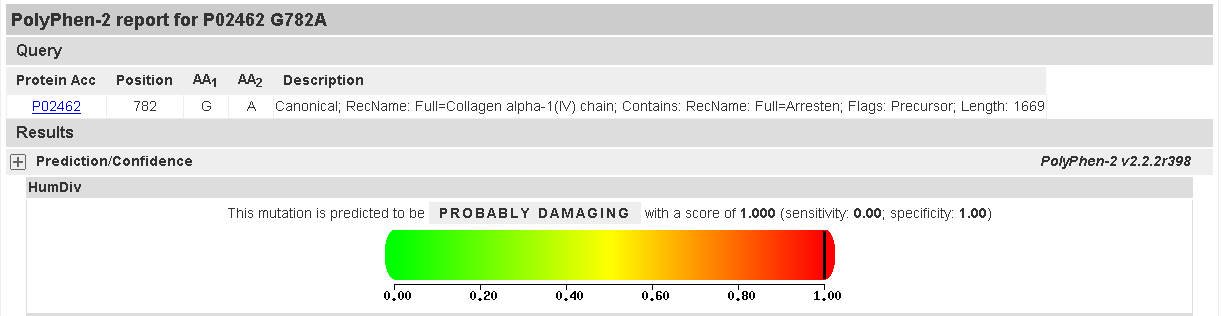
Figure. 1 The analysis result of SIFT Software

Figure. 2 The analysis result of Polyphen Software
